# Hygiene practices in slaughterhouses and public health risk: A scoping review

**DOI:** 10.1371/journal.pone.0334225

**Published:** 2025-11-03

**Authors:** Mohammad Abdus Shahid Hossain, Nelima Ibrahim, Karen N. Barnett

**Affiliations:** 1 Population Health and Genomics, University of Dundee, Scotland, United Kingdom; 2 Department of Livestock Services, Dhaka, Bangladesh; Federal Ministry of Agriculture and Rural Development, NIGERIA

## Abstract

**Introduction:**

Slaughterhouses are facilities where animals are slaughtered for meat production. Poor hygiene practices in these facilities, especially in developing countries, pose significant public health risks. The lack of regulation and substandard conditions can lead to the transmission of diseases from animals to humans, particularly affecting slaughterhouse workers who are in close contact with animals and animal products. A scoping review was conducted to map and summarize existing evidence on hygiene practices in slaughterhouses and evaluate their effectiveness in mitigating public health risks.

**Methodology:**

This review used the Joanna Briggs Institute Collaboration (JBI) scoping review inclusion framework Population Concept and Context (PCC) and PRISMA for Scoping Review (PRISMA-ScR) guidelines. Databases searched included; EBSCO Medline, Web of Science, EMBASE, and grey literature from BASE and Google Scholar. The studies were screened for eligibility and data extracted from included studies using the JBI data extraction chart. Included studies were assessed for risk of bias using cross-sectional and prevalence studies quality assessment tools.

**Results:**

Fourteen studies were included published between January 2002 and May 2023. These studies were conducted in 10 countries, with one-third (n = 5) originating from Nigeria. Twelve studies used cross-sectional surveys, while one employed a prevalence survey. This review identified common hygiene practices relating to personal hygiene [handwashing with soap and water] and environmental hygiene (cleaning tools and surfaces). Overall, workers’ reported that they had good knowledge of hygiene practices; handwashing (95.7%), using protective apparatus (89.7%), and cleaning equipment (89.3%). Studies reported that over half of slaughterhouse workers wore protective clothing. However, much less (12%) reported that they cleaned their knives before and after use, and it was reported that some slaughterhouses disposed of refuse improperly (bush], raising important public health concerns. Only three studies clearly linked slaughterhouse hygiene practices with public health risks.

**Conclusion:**

Improving hygiene practices in slaughterhouses is crucial for reducing public health risks. Gaps in knowledge, inadequate training, insufficient inspections and poor infrastructure were identified as key areas for improved guidelines and policy intervention.

## Introduction

Meat is a vital component of human nutrition, providing essential nutrients such as iron, B vitamins, and amino acids necessary for maintaining health [1]. The consumption of meat has a long history, but it also poses significant health risks due to potential contamination with pathogens during processing [2]. These pathogens can originate from various sources, including animals, human handlers, contaminated equipment, and water used in processing [3,4].

The concept of the slaughterhouse dates back to 1890, becoming a profitable venture until the First World War [5]. The development of slaughterhouses varies by country, influenced by factors such as social status, types of animals slaughtered, and cultural practices [6]. In developed nations, slaughterhouses are often large, industrialized facilities focused on meat packing [7,8].

Slaughterhouses can be categorized into four types based on regional, cultural, and religious factors [9]. These include modern facilities catering to high-value domestic and export markets, larger older government-owned establishments, small to medium-sized municipal or private slaughterhouses, and community-level slaughter “slabs.” Regulatory bodies authorize slaughterhouses for the sanitary slaughtering and processing of animals and the storage and preservation of meat products for human consumption [10].

Hygiene is essential in meat processing, defined as the practice of maintaining cleanliness to prevent illness [11,12]. The Food and Agriculture Organization (FAO) and World Health Organization (WHO) established the Food Standards Program to develop the Codex Alimentarius, a globally recognized set of food safety standards [13]. The Codex Alimentarius Code of Hygienic Practice for Meat (CHPM) emphasizes a risk-based approach to ensuring sanitary conditions throughout the meat production chain.

Effective sanitary practices are crucial in controlling contamination sources during meat processing in slaughterhouses. Foundational principles, such as those outlined by Skaarup [1985], emphasize the importance of proper facility design, including isolated locations, impermeable flooring, reliable water supplies, and effective drainage systems [14]. These principles have been further developed in contemporary guidelines—such as those by the FAO and EFSA, which underscore the importance of stringent hygiene monitoring systems and comprehensive biosecurity practices throughout the meat production and transport chain [15–17].

Key aspects of slaughterhouse hygiene include maintaining a separation between clean and dirty operations to prevent cross-contamination, as well as providing adequate lairage facilities to ensure humane treatment of animals and improve processing conditions. Modern standards highlight stress reduction in animals prior to slaughter as a critical factor for improving meat quality and minimizing contamination risks [18].

Critical slaughter processes, such as stunning, bleeding, and evisceration, must be executed with care to avoid contamination. While Skaarup, 1985 emphasized the importance of separate rooms for scalding, skinning, and other species-specific procedures, current guidelines recommend the use of automated systems and advanced disinfection technologies to enhance hygiene [14,16,19]. Additionally, chilling and freezing capacities are crucial for meat preservation, particularly in tropical climates, where temperature control is vital to prevent bacterial growth [17].

Environmental hygiene practices, including effective pest control, waste management, and fencing, remain essential. Modern recommendations further advocate for biosecurity measures, such as controlled entry points and footbaths, to minimize contamination risks [18]. Personal hygiene also plays a critical role, with regular training programs ensuring that workers adhere to updated food safety protocols [17]. These evolving practices reflect a longstanding concern for public health, as the relationship between meat consumption, animal slaughter, and disease prevention has been recognized for centuries, with regulatory efforts dating back to ancient Rome [20].

Several studies have highlighted the importance of sanitation practices in slaughterhouses and their impact on public health [29,30,38]. All existing research is conducted in a specific region or a country but not on a worldwide scale. The purpose of this review is to learn more about how sanitation standards in slaughterhouses affect public health risks globally. By broadening the existing knowledge base, this study seeks to compare hygiene practices across various countries and provide insights into adapting these practices to local contexts to mitigate public health risks. The findings will serve as a valuable resource for policymakers in developing countries and enforcing health-related legislation. Additionally, the review will help map existing literature and identify research gaps to guide future studies.

## Methodology

This review follows the Arksey and O’Malley [2005] approach [21], refined by the Joanna Briggs Institute [22,23], and adheres to JBI guidelines and the PRISMA-ScR framework (detailed in the supplementary files S1 Table) [24,25]. The aims of this scoping review are to map and summarize existing evidence on hygiene practices in slaughterhouses, and evaluate their effectiveness in mitigating public health risks.

### Eligibility criteria

Studies were included if they were published in English or available in an English-translated version, between January 1, 2002, and May 17, 2023, with full-text access and using any study design or methodology. Studies were excluded if the full text was not accessible, if they did not adhere to the PCC (Population, Concept, Context) framework, or if they represented secondary research such as reviews or meta-analyses.

#### Search strategy.

Several databases were searched including EBSCO Medline, Web of Science, and EMBASE, along with grey literature sources from BASE and Google Scholar. EBSCO Medline, a reliable resource from the National Library of Medicine, covers diverse fields including pre-clinical sciences, dental hygiene, and animal health. Web of Science was chosen for its extensive database and subject indexing, while Embase, part of the NHS Knowledge Network, provides comprehensive resources from over 100 vetted health and social care providers. Grey literature, which encompasses non-commercially published material from government, academic, business, and industry sources, was also incorporated to capture both published and unpublished articles not typically found in standard databases. A manual search of the reference lists from included articles was conducted to check for any additional eligible studies, not listed within the searched databases. The search was carried out over four weeks, concluding on May 18, 2023.

The PCC framework (Population, Concept, Context) was used to inform the research question. Population refers to slaughterhouse workers, Concept to hygiene practices, and Context to slaughterhouses in different countries. Search terms were based on Medical Subject Headings (MeSH) and keywords relating to Hygiene, Sanitation, and Public Health, combined using Boolean operators ‘AND’ and ‘OR’. The specific search terms included:

(Hygiene OR Sanitation OR Regimen OR Cleanliness) AND (slaughterhouse OR abattoir OR “meat processing plant” OR “slaughterhouse workers” OR “slaughterhouse staff” OR “abattoir workers” OR “abattoir personnel” OR “meat processor” OR “meat handler”) AND [“public health” OR “community health”).

Initial searches adhered only to the criteria of language (English) and publication date [01/01/2002 to 17/05/2023].

The Tables 1–3 below provides an example of the search methodology employed to identify articles across various databases using MeSH terms and key phrases. Additional search methods utilized are detailed in the supplementary files (S2–S4 Tables).

**Table 1:** The search strategy from EMBASE Ovid database.

Searched MeSH and text words		Results
1	hygiene/	44513
2	hygiene.mp.	140281
3	sanitation/	17140
4	sanitation.mp.	31969
5	regimen.mp.	325909
6	cleanliness.mp.	4912
7	1 OR 2 OR 3 OR 4 OR 5 OR 6	492364
8	slaughterhouse/	10612
9	slaughterhouse.mp.	12311
10	abattoir.mp.	4288
11	meat industry/	2950
12	meat processing plant.mp.	114
13	slaughterhouse worker.mp.	33
14	abattoir worker/	178
15	abattoir staff.mp.	6
16	abattoir personnel.mp.	12
17	8 OR 9 OR 10 OR 11 OR 12 OR13 OR 14 OR 15	16550
18	public health/	235099
19	public health.mp.	583037
20	community health.mp.	60973
21	18 OR 19 OR 20	629120
22	7 AND 17 AND 21	114

Footnote: a. mp. = all text.

**Table 2:** The search strategy from EBSCO Medline database.

Searched MeSH and text words		Results
1	Hygiene	144966
2	Sanitation	14897
3	Regimen	200531
4	Cleanliness	5161
5	1 OR 2 OR 3 OR 4	355462
6	Slaughterhouse	4997
7	Abattoir	5894
8	Meat processing plant	254
9	Slaughterhouse workers	251
10	Slaughterhouse staff	5
11	Abattoir workers	209
12	Abattoir personnel	19
13	Meat Processor	128
14	Meat Handler	66
15	6 OR 7 OR 8 OR 9 OR 10 OR 1 OR 12 OR 13 OR 14	8810
16	public health	1115574
17	community health	232479
18	16 OR 17	1239640
19	5 AND 15 AND 18	315

**Table 3:** The search strategy from Web of Science database.

Searched MeSH and text words		Results
1	Hygiene	378344
2	Sanitation	26863
3	Regimen	269658
4	Cleanliness	9510
5	1 OR 2 OR 3 OR 4	673041
6	Slaughterhouse	9411
7	Abattoir	6526
8	Meat processing plant	4638
9	Slaughterhouse workers	570
10	Slaughterhouse staff	368
11	Abattoir workers	474
12	Abattoir personnel	171
13	Meat Processor	983
14	Meat Handler	371
15	6 OR 7 OR 8 OR 9 OR 10 OR 1 OR 12 OR 13 OR 14	20563
16	public health	954955
17	community health	811668
18	16 OR 17	1610865
19	5 AND 15 AND 18	347

#### Selection of source of evidence.

**Fig 1:**
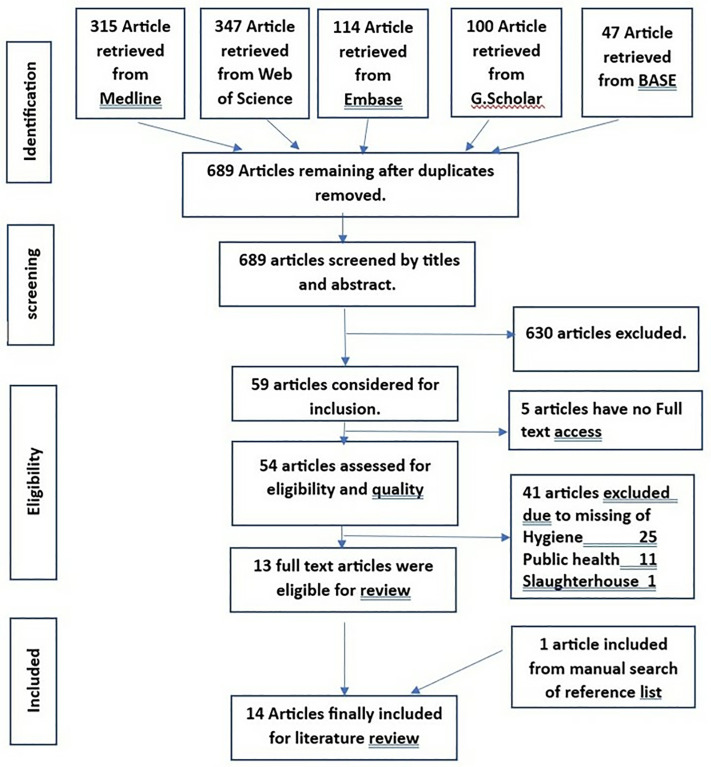
PRISMA flow diagram.

All included results were imported into EndNote 20, where duplicates were removed. Author [MH] screened titles and abstracts using the reviews inclusion/exclusion criteria. Full-texts were retrieved for all potentially relevant sources. MH reviewed the full-text articles for eligibility and documented reasons for excluding any sources that did not meet the criteria (Fig 1).

#### Extraction of data.

The data extraction process, referred to as “data charting”, was used to extract relevant data from each study to answer the review’s objectives [26]. A charting table was created to capture essential details from each source, including the author, publication year, country of origin, aims, sample size and population [if applicable], methodology, type of intervention and results. Key findings related to the review’s questions were also captured. The extracted data were recorded in a Microsoft Excel ‘data extraction chart’.

#### Quality assessment.

Quality was assessed using the JBI critical appraisal tools (CAT) (2020) [27]. The checklist by CAT specific to each study design was used. Most studies were cross-sectional, with one prevalence study. Cross-sectional studies were evaluated using the Analytical Cross-Sectional Studies checklist, while the prevalence study used its respective checklist. Studies were rated based on their responses, with “yes,” “no,” “unclear” and “not applicable” options.

#### Data synthesis and reporting.

Data from each study were evaluated and synthesized to address the research question. The focus was on public health threats and hygiene practices in slaughterhouses.

Data were tabulated or charted with narrative summaries of the review findings based on study design, geographical location, publication year, participant type, hygiene practices, and their impact on public health. A narrative synthesis of the main findings is provided.

## Results

### Selection of source of evidences

EMBASE Ovid database yielded 114 articles, EBSCO Medline database 315 articles, and Web of Science 345 articles. BASE and Google Scholar were searched for grey literature, resulting in 147 relevant articles. A total of 923 results were identified and imported into EndNote 20, where 234 duplicates were removed. The remaining 689 articles were screened based on titles and abstracts. Of these, 630 articles did not meet the eligibility criteria, leaving 59 articles for full-text review. Full-texts were unavailable for five articles. Forty one articles were excluded for not sufficiently addressing key aspects such as hygiene practices, public health, and/or slaughterhouses. The reasons for exclusion are provided in S3. In total, 13 full-text articles were identified following eligibility screening. An additional article was identified through manual reference list searches, bringing the total number of studies included in the review to 14. The article selection process is illustrated in Fig 1 (PRISMA-ScR) [25].

### Study characteristics

The review included a total of fourteen studies that focused on slaughterhouse hygiene practices and their impact on public health. The characteristics of these studies were organized based on; geographic context, publication year, study design, survey population/details, and outcome measures (Table 4).

**Table 4:** Summary of individual study characteristics.

Author/s, year Reference	Country	Study type	Study population	Key findings
Al Banna et al. 2021 [28]	Bangladesh	Cross-sectional Study by impersonal interview	Meat handlers 300	Worker knew about hand washing before functioning (95.7%), wearing gloves, aprons, and headwear (89.7%), and correctly cleaning the tools (89.3%).
Laban et al. 2021 [29]	Egypt	Cross-sectional Study by questionnaire, inspection and lab test	Worker 45 swab sample 91 water sample 5, beef sample 36	In slaughterhouse 1, 67% of the workers said they practised cleaning and disinfection compared to 3% in slaughterhouse 2 and 0% in slaughterhouse 3. Swabs taken from the floor during a microbiological test revealed that the highest levels of bacteria and coliforms.
Bahiru et al. 2022 [30]	Ethiopia	Cross-sectional Study by lab test and interview	Worker 113 Sample of sputum 54	One-third of the workers (32.74%) at the slaughterhouse claimed to have cleaned their hands after completing the activity. Four (7.41%) of the 54 samples of sputum taken from slaughterhouse workers tested positive for the growth of mycobacteria.
Islam et al. 2022 [31]	India	Cross-sectional Study by interview, observation and discussion	Butcher 60	Most respondents (88.33%) did not wear safeguards such as gloves or a face mask. In addition to the fact that nearly two-thirds (65%) did not wash their hands before and after handling meat. Most butchers (88.33%) did not clean their knives before and after cutting meat.
Cook et al. 2017 [32]	Kenya	Cross-sectional Study by interview and observation	Workers 738	Half of the workers (n = 369) used personal protective equipment, including 53% of those who wore protective coats and 49% of those who wore rubber boots. Almost one-third of the workers knew about zoonotic diseases, and 18% of the workers agreed to slaughtering animals they thought were ill or diseased.
Edet O 2022 [33]	Nigeria	Cross-sectional Study by interview and observation	Slaughterhouse 3	A total of 66.7% of the slaughterhouses dispose of their waste adjacent to bush, while 33.3% engage in open burning. In the same way, all slaughterhouses (100%) poured their waste into public sewers whenever it came to dealing with wastewater.
Alhaji et al. 2015 [34]	Nigeria	Cross-sectional Study by questionnaire	Worker 385 age 20+	The most frequent preventive practise was cleaning the cutting surface and utensils (17.4%), followed by using hand gloves (16.5%). About 33.4% of the workers were familiar with incineration, 30.5% with burial, and 9.3% with the processing of by products.
Agu et al. 2021 [35]	Nigeria	Cross-sectional Study by interview	Workers 188	75.5% of worker had adequate knowledge, and 85.6% had appropriate hygienic habits. But 44.1% of people wash their hands before and after handling meat, and 45.2% clean their work surfaces with soap and water.
Njoga et al. 2023 [36]	Nigeria	Cross-sectional Study by observation, PMI and questionnaire	Workers 157	About 71.3% of the slaughterhouse workers processed carcasses on bare floor, 52.2% used same bowl of water to wash multiple carcasses. While 72% did not wear personal protective equipment during meat/carcass processing. There were Several gross lesions indicating of porcine cysticercosis, infectious bovine pleuro-pneumonia, fascioliasis, and bovine tuberculosis.
Richard et al. 2015 [37]	Nigeria	Cross-sectional Study by interview and observation	Workers 11	Only two people (18.2%) who treat slaughterhouse waste wear personal protection equipment (lab coats and rain boots). The slaughter hall’s floor was used for all slaughter procedures. It was noticed that some animal body parts were being removed from the slaughterhouse in a very unsanitary condition, spreading blood on the ground outside the slaughterhouse.
Almasri et al. 2019 [38]	Saudi Arabia	Prospective cohort study by interview and lab test	Male workers 80 blood sample 80	About 96.25% (n = 77) of the employees washed their hands with soap and water, and 98.75% (n = 79) did not wear eye protection. Very few of them used aprons and only 15% (n = 12) of them regularly utilised surgical masks. Six butchers tested positive for Brucella and one tested positive for Alkhurma Hemorrhagic Fever (AHF).
Qekwana et al. 2017 [39]	South Africa	Cross-sectional Study by interview	Workers 105	Around 62.64% of the respondents wore protective clothing while Only 12.50% of the participants washed their cutting edges before slaughtering and trimming. Most people (84.3%) used the same knife throughout the slaughtering procedure and cleaned when it got dirty.
Jeffer et al. 2021 [40]	Tanzania	Cross-sectional Study by interview		95% of employees working in butcher shops and supermarkets frequently wash their hands. However, they do not use gloves. Environmental hygiene, such as the cleanliness of the floor and the meat storage area, was satisfactory.
Komba et al. 2012 [41]	Uganda	Prevalence study By PMI (follow up) and interview	Cattle 30,713	Complete carcasses, lungs, livers, hearts, and heads were all rejected because of zoonotic disease included bovine tuberculosis, cysts from Cysticercus bovis, fasciolosis, and hydatidosis. The entire carcass dressing process was carried out manually on the ground. Because of water scarcity, the floor wasn’t consistently cleaned with water.

The studies included in this review were conducted across 10 different countries, with one-third [n = 5] carried out in Nigeria. Each of the remaining countries contributed one study. All studies were published between January 1, 2002, and May 17, 2023, with the most recent published on March 3, 2023. The review encompassed three study designs, with almost (n = 12) being cross-sectional studies, while there were one cohort and another prevalence study included.

#### Sample size of included studies.

The reviewed studies, focusing on diverse contexts, including butcher shops, slaughterhouses and super markets, as well as occupational groups such as slaughterhouse workers, meat handlers and butchers, as well as microbiological samples. The samples sizes varied considerably, with the number of slaughterhouse ranging from 3 to 196, study participants from 11 to 738 and microbiological or lab sample were 5 to 30713, respectively, to assess hygiene practices linked to public health importance.

#### Outcome measures.

The most frequently reported outcomes were related to both personal and environmental hygiene practices [n = 11]. All outcomes are detailed below (Table 5).

**Table 5:** Outcomes of the studies.

Variable	Outcome	References
Personal Hygiene Practice	Uses of protective hand gloves, aprons, goggles, face mask, rubber boot, hand wash and soap/sanitizer	[28,30–35,37–40]
Environmental Hygiene	Cleaning of cutting materials, dumping of waste, sanitary facilities of slaughterhouse	[29,31–37,39–41]
Public Health	Tuberculosis, salmonellosis, rift valley fever, Alkhurma Hemorrhagiic Fever, fasciolosis and hydatidosis, porcine cysticercosis	[28–30,32,36,38,41]

### Critical appraisal and risk of bias

All the studies were evaluated for quality and risk of bias using the JBI Critical Appraisal Tools (CAT). Twelve of the fourteen studies were assessed using the analytical cross-sectional studies checklist, while the remaining two studies were evaluated using the cohort and prevalence studies checklist [24]. The quality assessment identified eleven high-quality studies, with the remaining three categorized as medium-quality (Table 6). Using the JBI critical appraisal check list articles were given a score of ‘1’ for yes and ‘0’ for all other responses. The total score is measured by percentage where a score of more than 70% counts as high quality, 50–70% for medium quality and less than 50% low quality. A high score indicates a low risk of bias. Three studies were scored as medium quality. Among these studies, one included a sampling error, one did not clearly identify possible confounding and one reported unreliable measurement of a study exposure.

**Table 6:** Quality assessment ratings of studies using JBI CAT tools.

CAT checklist	Author/reference	CAT quality assessment					
		Yes	No	Unclear	Not applicable	Score (%)	Quality
Analytical Cross-sectional study	Al Banna et al., 2021 [28]	6	1	1		75	High
	Laban et al., 2021 [29]	5	1	1	1	62.5	Medium
	Bahiru et al., 2022 [30]	5	2	1		62.5	Medium
	Islam et al., 2022 [31]	6	1		1	75	High
	Cook et al., 2017 [32]	6	1		1	75	High
	Edet, 2022 [33]	6	1		1	75	High
	Alhaji and Baiwa, 2015 [34]	6	1	1		75	High
	Agu et al., 2021 [35]	7			1	87.5	High
	Njoga et al., 2023 [36]	5		2	1	62.5	medium
	Richard et al., 2015 [37]	6		1	1	75	High
	Qekwana et al., 2017 [39]	6		2		75	High
	Jeffer et al., 2021 [40]	6		1	1	75	High
Cohort study	Almasri et al., 2019 [38]	8	1		2	72.7	High
Prevalence study	Komba et al., 2012 [41]	7		2		77.7	High

### Synthesis/Summary of Results

#### Knowledge of hygiene and zoonotic risks.

Several studies reported varying levels of knowledge among slaughterhouse workers regarding hygiene practices and zoonotic diseases. In Bangladesh, 95.7% of meat handlers knew the importance of handwashing, while 89.7% were aware of using protective gear like gloves and aprons [28]. In Nigeria, 75.5% of workers demonstrated adequate knowledge, and 39.2% recognized that improper practices could harm public health [35]. Similarly, more than 50% of workers in a Nigerian study understood zoonotic disease transmission [36]. However, in Ethiopia, 63.7% of respondents were unaware of the role of hot water, detergents, and sanitizers in hygiene maintenance [30].

#### Attitudes towards hygiene and safety.

Attitudes towards hygiene varied. Despite high levels of knowledge, poor attitudes were often observed. For instance, in Bangladesh, nearly half of the workers admitted to eating, drinking, or smoking in processing areas [28]. In Nigeria, a significant proportion of workers acknowledged the importance of hygiene but failed to adopt consistent practices [34,35]. Similarly, workers in Uganda expressed concern about hygiene but did not use gloves or PPE consistently [40].

#### Hygiene practices and personal protective equipment (PPE) use.

The actual practices often fell short of knowledge levels:

**Use of PPE:** PPE usage was alarmingly low in several countries. In India, 88.3% of workers did not use gloves or masks [31], and in Nigeria, some studies reported no use of protective gear at all [33,37]. In contrast, higher compliance was seen in Saudi Arabia (98.75% wore PPE) [38] and South Africa (62.6%) [39].

**Handwashing and Cleaning Tools:** Hand hygiene was inconsistently practiced. While 96.25% of workers in Saudi Arabia washed hands with soap [38], only 32.7% did so in Ethiopia [30], and 65% of Indian butchers skipped handwashing entirely [31]. Tool hygiene was similarly neglected: in South Africa, only 12.5% cleaned cutting tools before use [39].

**Infrastructure and Facility Conditions:** Basic infrastructure varied across regions. In Kenya, 65% of slaughterhouses had walls and cement floors, but only 20% had handwashing stations [32]. Nigerian slaughterhouses often lacked proper waste disposal systems, with waste dumped in bushes or openly burned [33]. In Tanzania, floors were rarely cleaned due to water scarcity [41].

**Meat Handling Practices:** Several studies reported unsafe practices such as processing meat on bare floors (e.g., 71.3% in Nigeria) [36] and using the same knife throughout the process without cleaning (84.3% in South Africa) [39].

#### Zoonotic disease detection and risk.

Biological testing in some studies revealed zoonotic risks. In Ethiopia, 7.4% of sputum samples from workers tested positive for Mycobacteria [30]. In Saudi Arabia, serological tests identified six cases of Brucella and one case of Alkhurma Hemorrhagic Fever among workers [38]. Tanzania reported frequent meat rejections due to zoonoses such as bovine tuberculosis and hydatidosis [41].

The reviewed studies consistently reveal critical shortcomings in hygiene management within slaughterhouses across low- and middle-income countries (LMICs). Personal hygiene practices, such as regular handwashing and tool disinfection, were poorly adhered to in many settings, with compliance rates ranging from under 35% to over 90%, depending on the country and facility. The use of personal protective equipment (PPE) was similarly inconsistent—some facilities reported no PPE use at all, while others showed moderate adoption, often without full compliance. Environmental sanitation was often inadequate, with many abattoirs lacking proper waste disposal systems, cleanable surfaces, and reliable water access. Several studies also reported microbiological or serological evidence of zoonotic pathogens, including Brucella, Mycobacterium, and Alkhurma hemorrhagic fever virus, indicating substantial occupational health risks. Overall, the most common barriers to safe practices included limited knowledge, insufficient infrastructure, poor enforcement of safety protocols, and lack of ongoing training for slaughterhouse workers.

## Discussion

This scoping review maps and summarizes the existing evidence on hygiene practices in slaughterhouses and attempts to evaluate their effectiveness in mitigating public health risks. We identified 14 studies conducted predominantly in Nigeria between 2015 and 2023 [33–37]. Most of these studies were cross-sectional surveys, with the exception of one prospective cohort study and one prevalence study [41].

Eleven studies focused on personal hygiene [28,30–35,37–40], 11 on environmental hygiene [29,31–37,39–41] and seven on public health implications [28–30,32,36,38,41]. Additionally, about one-third of the studies explored workers’ knowledge of hygiene, sanitation, and zoonotic diseases [28,30,32,34–36] while only three [34–36] studies made positive association between hygiene practices and knowledge.

One-third [n = 5] of the included studies focused on hand washing practices among slaughterhouse workers, and the findings on hand washing of the included studies were unsatisfactory. Only two studies reported that most workers consistently washed their hands with soap and water. In Ethiopia [38,40], Teferi, 2022 found that 88.8%, 89.3%, and 88.4% of meat handlers washed their hands after handling waste, using the toilet, and before and after handling meat, respectively [42]. However, Upadhayaya and Ghimire, 2018 noted that 98.42% of meat handlers did not wash their hands after handling potentially infected meat, with those who did only using water and soap [43]. Inadequate hand washing facilities in slaughterhouses remain a significant public health risk [32].

The included studies highlighted insufficient use of protective apparatus, such as aprons, gloves, and masks, among slaughterhouse workers. Five studies reported limited or no use of protective equipment during slaughter [28,31,36,38,40]. Proper protective clothing helps prevent contamination and zoonotic diseases like brucellosis [44]. The lack of hygienic practices in food processing and animal husbandry affects farmers, butchers, hunters, veterinarians, and laboratory staff. Key preventive measures include using protective barriers and proper handling of carcasses and internal organs. However, insufficient knowledge, limited access to protective materials, inadequate training, poor enforcement of slaughter regulations, and lack of experience contribute to the inadequate use of protective apparatus [28–31,34–38].

In order to produce healthy meat, slaughterhouses must follow standard operating procedures and maintain basic environmental and sanitary conditions [45]. Environmental hygiene in slaughterhouses is significantly affected by infrastructure, protective apparatus, and workers’ commitment to sanitation. Several studies reported that slaughterhouse workers often used bare floors for slaughtering, decapitating, and eviscerating, which led to high bacterial loads due to insufficient cleaning and water shortages [29,36,37,41]. Poor drainage systems were highlighted in three studies, with open drains causing waste and blood accumulation, which fostered maggot growth [33,36,41].

Sanitation practices, including tool cleaning and waste disposal, were unsatisfactory in several studies due to low hygiene awareness and training [33–35]. However, Jeffer et al. [2021] reported improved hygiene where regular inspections by veterinarians ensured better practices [40]. Water shortages hindered carcass washing and equipment sterilization, while improper cleaning practices raised concerns about meat contamination [31,39].

Only 45.2% and 17.4% of workers reported using soap and water to sanitise their work surfaces [35] and cutting surface and utensils [34] respectively. Another study found that 65% of slaughterhouses had basic infrastructure like walls and cement floors, while 60% had toilets and only 20% had hand-washing facilities [32]. This study also showed, the slaughterhouse were working without authority and regulations. Among 66% of slaughterhouses the water source was a borehole with water carried by hand., Dogs, an environmental threat were observed in 78% of the slaughterhouses, with rats observed in a much smaller number.

The reviewed studies raised significant public health concerns related to food safety in slaughterhouses. Islam et al., 2022 found that 98.33% of workers spread blood over carcasses to make meat appear fresher, increasing the risk of microbial contamination [31]. In southeastern Nigeria, zoonotic diseases like tuberculosis and cysticercosis were found in carcasses, indicating poor postmortem inspections [36].

Cook et al. [2017] reported tuberculosis, typhoid, and malaria among slaughterhouse workers, partly due to inadequate handwashing facilities (20%) and a lack of toilets (40%) [32]. Al Banna et al., 2021 highlighted rejection rates of carcasses due to zoonotic diseases, with 0.026% of whole carcasses 1.96% of lungs, 1.61% of livers, 0.02% of hearts and 0.21% of heads being rejected [28]. Additionally, 64.3% of workers handled meat while having cuts or injuries, raising zoonotic risks.

Serological tests showed six butchers tested positive for Brucella, and only 15% of workers regularly used face masks [38]. Laban et al. [2021] found high bacterial counts on slaughterhouse floors [29], while Bahiru et al. [2022] reported 7.31% Mycobacteria growth in workers’ sputum samples [30]. Zoonotic diseases like hydatidosis and bovine tuberculosis led to carcass condemnations [41].

The included studies reported varying levels of hygiene knowledge among slaughterhouse workers. Alhaji et al., 2015 reported that 39.2% of workers were aware of the public health impacts of incomplete slaughter activities, and 33.4% had experience with incineration, while 30.5% had experience with burial methods [34]. Workers with better knowledge of proper waste disposal tended to follow better preventive practices [34].

In contrast, Bahiru et al., 2022 found that 63.71% of workers were unaware of using hot water, detergent, and sanitizers for hygiene [30]. Meanwhile, 50% understood zoonotic disease transmission [36], and 75.5% had adequate hygiene knowledge [35].

Although one study reported that 95.7% of workers recognized the importance of handwashing and wearing protective apparatus [28], Islam et al., 2022 found that 65% admitted they did not wash their hands before and after handling meat [31]. Education levels played a key role in hygiene knowledge, with three-fourths of respondents only having an eighth-grade education, contributing to poor practices [34]. This study also reported that only 27.8% and 29.4% of workers knew about hygiene and sanitation respectively.

Three studies identified in this review explored the association between workers’ knowledge and hygiene practices. One study found a significant association (P < 0.05) between knowledge and preventive actions like sanitation [34], noting that younger, unmarried, government, and female workers had poorer knowledge and associated poorer hygiene practices. Another study found that workers with good hygiene knowledge were five times more likely to follow proper hygiene practices [35].

The third study highlighted a significant correlation between education and PPE use (P = 0.022) but no association to use of same bowl of water (P = 0.29). Though workers with tertiary education and over 20 years of experience were more likely to use PPE to prevent zoonotic diseases [36].

Laban et al. [2021] linked contamination levels in slaughterhouses to poor hygiene practices [29]. Slaughterhouse named 3, one of the study point had the highest bacterial count from swabs of walls, knives, workers’ hands, and hooks, except for the floor in slaughterhouse 1. This was attributed to improper knife use and irregular cleaning. Food handlers can spread bacteria during processing, leading to foodborne illnesses [46,47].

Bahiru et al., 2022 found that 92.92% of workers consumed raw meat or milk, increasing the risk of tuberculosis, particularly among younger workers (aged 18–47). This disease primarily affects adults in their most productive years, though all age groups are at risk [48]. Tuberculosis is transmitted through airborne particles from an infected person [49].

Although several studies did not establish a direct link between hygiene and public health, they emphasized the importance of proper practices for worker safety [36,41]. Most studies were conducted in low-income countries with weak sanitation enforcement, in contrast to stricter regulations in developed countries [37].

The findings of this review highlight the need for several key actions to improve meat safety and protect public health. First, it is crucial that employees in abattoirs receive training in hygienic practices based on Hazard Analysis and Critical Control Point (HACCP) principles [45], which have been shown to improve food safety standards [40]. Government regulatory authorities should also take action to upgrade infrastructure and food safety systems to ensure that standards are consistently met [31]. To mitigate risks associated with poor hygiene practices, implementing a food safety management program is essential, as it has been proven to reduce contamination and ensure safer meat production [39]. Additionally, training programs should be tailored to provide workers with a deeper understanding of standard abattoir operations, focusing on meat quality and environmental health [34]. It is also important for the Food Safety Authority to mandate that meat handlers complete formal training in order to obtain a license, though the industry’s current use of such practices needs further exploration [28]. Strict enforcement of food safety laws is necessary to enhance meat quality, food safety, and, ultimately, public health [36]. Furthermore, operating abattoirs in line with recognized international standards will help ensure consistent practices across countries [37]. Finally, developing a One Health strategy for disease surveillance, which benefits employees, producers, and consumers, is recommended as a comprehensive approach to food safety [32]. These recommendations should be seen as a step toward strengthening both food safety and public health by addressing the gaps identified in the review, ensuring that the meat production process adheres to the highest standards.

Over half of the included studies had large sample sizes (>100 participants), enhancing reliability [28,30,32,34–36,39–41]. One-third of the studies had a small sample size (<100), reducing the generalizability of their findings [29,31,33,37,38,50]. Many studies used self-administered questionnaires, which might have introduced reporting bias [28–40]. Additionally, some studies did not include temporary workers, leading to potential sampling bias [38]. Although systematic scoping reviews typically involve at least two reviewers [22], the constraints of a student project precluded the recruitment of a second reviewer.

## Conclusion

Findings from this scoping review highlighted various links between slaughterhouse hygiene standards and public health risks. Key issues include a lack of awareness, inadequate training in proper hygiene, inconsistent meat inspections [both antemortem and postmortem], and poor abattoir infrastructure and poor regulations. Additionally, the absence of necessary tools and equipment, along with limited commitment from food safety authorities to enforce existing legislation, significantly impacts meat quality and public health protection. These findings underscore the urgent need for stronger policies, comprehensive legislation, and effective regulatory frameworks to ensure consistent hygiene standards. Further research, particularly long-term follow-up studies, is recommended to identify previously unexplored aspects of hygiene practices and their associated public health risks.

## Supporting information

S1 Table Preferred Reporting Items for Systematic reviews and Meta-Analyses extension for Scoping Reviews (PRISMA-ScR) Checklist. (DOCX)

S2 Table JBI critical appraisal checklist for studies reporting prevalence data. (DOCX)

S3 Table JBI critical appraisal checklist for analytical cross sectional studies. (DOCX)

S4 Table Excluded articles with reasons. (DOCX)
