# Supplementary material for: Hygiene practices in slaughterhouses and public health risk: A scoping review
Source: PLoS One. 2025 Nov 3;20(11):e0334225. doi: 10.1371/journal.pone.0334225 (PMC12582449; doi:10.1371/journal.pone.0334225)
Supplement: S2 Table — (DOCX) [file pone.0334225.s002.docx]

**S2 Table. JBI critical appraisal checklist for studies reporting prevalence data**

|  | **Yes** | **No** | **Unclear** | **Not applicable** |
| --- | --- | --- | --- | --- |
| 1. Was the sample frame appropriate to address the target population? | □ | □ | □ | □ |
| 1. Were study participants sampled in an appropriate way? | □ | □ | □ | □ |
| 1. Was the sample size adequate? | □ | □ | □ | □ |
| 1. Were the study subjects and the setting described in detail? | □ | □ | □ | □ |
| 1. Was the data analysis conducted with sufficient coverage of the identified sample? | □ | □ | □ | □ |
| 1. Were valid methods used for the identification of the condition? | □ | □ | □ | □ |
| 1. Was the condition measured in a standard, reliable way for all participants? | □ | □ | □ | □ |
| 1. Was there appropriate statistical analysis? | □ | □ | □ | □ |
| 1. Was the response rate adequate, and if not, was the low response rate managed appropriately? | □ | □ | □ | □ |
