# Supplementary material for: Hygiene practices in slaughterhouses and public health risk: A scoping review
Source: PLoS One. 2025 Nov 3;20(11):e0334225. doi: 10.1371/journal.pone.0334225 (PMC12582449; doi:10.1371/journal.pone.0334225)
Supplement: S3 Table — (DOCX) [file pone.0334225.s003.docx]

**S3 Table. JBI critical appraisal checklist for analytical cross sectional studies**

|  | **Yes** | **No** | **Unclear** | **Not applicable** |
| --- | --- | --- | --- | --- |
| 1. Were the criteria for inclusion in the sample clearly defined? | □ | □ | □ | □ |
| 1. Were the study subjects and the setting described in detail? | □ | □ | □ | □ |
| 1. Was the exposure measured in a valid and reliable way? | □ | □ | □ | □ |
| 1. Were objective, standard criteria used for measurement of the condition? | □ | □ | □ | □ |
| 1. Were confounding factors identified? | □ | □ | □ | □ |
| 1. Were strategies to deal with confounding factors stated? | □ | □ | □ | □ |
| 1. Were the outcomes measured in a valid and reliable way? | □ | □ | □ | □ |
| 1. Was appropriate statistical analysis used? | □ | □ | □ | □ |
