# Supplementary material for: Hygiene practices in slaughterhouses and public health risk: A scoping review
Source: PLoS One. 2025 Nov 3;20(11):e0334225. doi: 10.1371/journal.pone.0334225 (PMC12582449; doi:10.1371/journal.pone.0334225)
Supplement: S4 Table — (DOCX) [file pone.0334225.s004.docx]

**S4 Table. Excluded articles with reasons**

| **Author/s**  **Year** | **Name of the Articles** | **Reason/missing for exclusion** |
| --- | --- | --- |
| Shamsul, et al. 2016 | Assessment exposure to Escherichia coli among abattoir workers in Malaysia | Full text |
| Abunna, et al. 2022 | Assessment of Food Safety Knowledge, Attitudes, and Practices among Meat Handlers in Bishoftu City, Ethiopia | Full text |
| Elblla 2018 | Assessment of Meat Safety Knowledge, Attitudes and Practices Among the Slaughterhouse Workers in ED Dueim City_ White Nile State | Full text |
| Ozdemir et al. 2010 | Determination of microbial surface contamination on beef carcasses | Full text |
| Vieira et al. 2014 | Game meat hygiene and safety in Portugal | Full text |
| Nastasijevic et al  2023 | Abattoir hygiene | Public health |
| Aynewa et al.  2021 | Assessment of Bacteriological Quality of Sheep Carcasses, Effect Level of 2.5% Citric Acid Spray on Bacterial Contamination of Meat, and Hygiene Practices of Workers in a Selected Abattoir in Debre Zeit Town, Central Ethiopia | Public health |
| Rejab et al.  2012 | Campylobacter in chicken carcasses and slaughterhouses in Malaysia | Public health |
| Al-Abd M  2022 | Evaluation of hygienic status of local slaughterhouses in Al – Marj, Libya and Its effect on microbial Load of Meat | Public health |
| Bhandari et al.  2022 | Factors associated with meat hygiene practices among meat-handlers in Metropolitan City of Kathmandu, Nepal | Public health |
| Abdullahi et al.  2016 | Food safety knowledge, attitude, and practice toward compliance with abattoir laws among the abattoir workers in Malaysia | Public health |
| Teferi SC  2022 | Food Safety Practice and Its Associated Factors among Meat Handlers in North Shewa Zone, Oromia, Ethiopia | Public health |
| Robinson et al.  2011 | Occurrences of thermophilic Campylobacter in pigs slaughtered at Morogoro slaughter slabs, Tanzania | Public health |
| Jessica et al.  2016 | Self-reported occupational injuries among industrial beef slaughterhouse workers in the Midwestern United States | Public health |
| Upadhayaya et al.  2018 | Survey on Good Hygiene Practices in Retail Meat Shops in Butwal Municipality, Nepal | Public health |
| Trajkoska et al.  2019 | The impact of the hygiene in the slaughterhouse on microbiological status of lamb carcasses | Public health |
| Ahmed et al.  2023 | Food Safety Programs that should be Implemented in Slaughterhouses: Review | Review |
| Lassok et al.  2023 | From Pig to Pork: Methicillin-Resistant Staphylococcus aureus in the Pork Production Chain | Review |
| Obidiegwu et al.  2019 | Public Health Challenges in Somachi Main Abattoir Owerri, Nigeria: A Review and Field Activity Report | Review |
| Ibrahim et al.  2021 | Role of slaughter facilities management in zoonoses and safety of meat produced for human consumption in Nigeria: a review | Review |
| Nyokabi et al.  2023 | From farm to table: exploring food handling and hygiene practices of meat and milk value chain actors in Ethiopia | Slaughterhouse |

| Pacholewicz et al  2015 | A comparison of fluctuations of Campylobacter and Escherichia coli concentrations on broiler chicken carcasses during processing in two slaughterhouses | Hygiene absent |
| --- | --- | --- |
| Manafi et al  2020 | Antibiotic resistance and biofilm formation ability of Salmonella serotypes isolated from beef, mutton, and meat contact surfaces at retail | Hygiene absent |
| Savin et al  2021 | Antibiotic‑resistant bacteria, antibiotic resistance genes, and antibiotic residues in wastewater from a poultry slaughterhouse after conventional and advanced treatments | Hygiene absent |
| Zhu et al.  2017 | Antimicrobial resistance and resistance genes in Salmonella strains isolated from broiler chickens along the slaughtering process in China | Hygiene absent |
| Okpala et al.  2021 | Assessing Nigerian Butchers’ Knowledge and Perception of Good Hygiene and Storage Practices: A Cattle Slaughterhouse Case Analysis | Hygiene absent |
| Blagojevic et al.  2013 | Assessment of potential contribution of official meat inspection and abattoir process hygiene to biological safety assurance of final beef and pork carcasses | Hygiene absent |
| Nyamakwere et al.  2016 | Assessment of salmonella, Escherichia coli, Enterobacteriaceae and aerobic colony counts contamination levels during the beef slaughter process | Hygiene absent |
| Abed et al.  2021 | Assessment of the work conditions of small slaughterhouses in the Thi-Qar governorate in Iraq | Hygiene absent |
| Rodrigues et al. 2018 | ATP-Bioluminescence and Conventional Microbiology for Hygiene Evaluation of Cutting Room Surfaces in Poultry Slaughterhouse | Hygiene absent |
| Katale et at.  2012 | Bovine tuberculosis at the human-livestock-wildlife interface: Is it a public health problem in Tanzania? A review | Hygiene absent |
| Rasschaert et al.  2020 | Campylobacter contamination of broilers: the role of transport and slaughterhouse | Hygiene absent |
| Kechih et al.  2018 | Carriage Methicillin-Resistant Staphylococcus aureus in Poultry and Cattle in Northern Algeria | Hygiene absent |
| Um et al.  2016 | Comparison of the incidence of pathogenic and antibiotic-resistant Escherichia coli strains in adult cattle and veal calf slaughterhouse effluents highlighted different risks for public health | Hygiene absent |
| Projahn et al.  2019 | Contamination of chicken meat with extended-spectrum beta-lactamase producing- Klebsiella pneumoniae and Escherichia coli during scalding and defeathering of broiler carcasses | Hygiene absent |
| Biasino et al.  2018 | Correlation between slaughter practices and the distribution of Salmonella and hygiene indicator bacteria on pig carcasses during slaughter | Hygiene absent |
| Hdaifeh et al.  2020 | Critical Analysis of Pork QMRA Focusing on Slaughterhouses: Lessons from the Past and Future Trends | Hygiene absent |
| Felin et al.  2016 | Current food chain information provides insufficient information for modern meat inspection of pigs | Hygiene absent |

| Takeshi et al.  2009 | Detection of Salmonella spp. Isolates from Specimens due to Pork Production Chains in Hue City, Vietnam | Hygiene absent |
| --- | --- | --- |
| Asakura et al.  2021 | Development and Evaluation of Fluorescence immunochromatography for Rapid and Sensitive Detection of Thermophilic Campylobacter | Hygiene absent |
| Melero et al.  2019 | Distribution and Persistence of Listeria monocytogenes in a Heavily Contaminated Poultry Processing Facility | Hygiene absent |
| Bello et al.  2015 | Management of slaughterhouses in northern Nigeria and the safety of meat produced for human consumption | Hygiene absent |
| Asare  2020 | Meat handling knowledge, attitudes, and practices among slaughterhouse workers in tamale metropolis | Hygiene absent |
| Meloni et al.  2017 | Occurrence of food-borne pathogens and process hygiene indicators in three Italian poultry slaughterhouses | Hygiene absent |
| Zakeri et al.  2023 | Prevalence and risk factors associated with Q fever infection in slaughterhouse workers in Fars province, Iran | Hygiene absent |
| Abukhattab et al.  2022 | Towards a One Health Food Safety Strategy for Palestine: A Mixed-Method Study | Hygiene absent |
